# Attachment-informed mental healthcare systems as ‘organisational caregivers’: ideas for the future

**DOI:** 10.1192/bjb.2024.94

**Published:** 2025-08

**Authors:** Alberto Salmoiraghi, Nicolò Zarotti

**Affiliations:** 1 Betsi Cadwaladr University Health Board, Bangor, UK; 2 Bangor University, Bangor, UK; 3 Wrexham University, Wrexham, UK; 4 Department of Clinical Neuropsychology, Manchester Centre for Clinical Neurosciences, Salford Royal NHS Foundation Trust, Salford, UK; 5 Division of Health Research, Lancaster University, Lancaster, UK

**Keywords:** Mental health services, psychosocial interventions, patients, community mental health teams, psychological medicine

## Abstract

Over the past 50 years, mental health services have evolved significantly, influenced by shifts in theoretical and practical approaches to mental disorders. Key among these changes are the biopsychosocial and recovery models, which highlight resilience and quality of life in treatment. However, traditional psychiatry has often struggled to embrace these changes because of reductionist perspectives that overlook psychosocial factors, resulting in fragmented care and reduced accessibility. Proposed solutions have faced implementation barriers in absence of a coherent theoretical framework. Here, we outline how attachment theory may offer a promising framework to drive systemic change in mental health by emphasising secure emotional bonds at both the organisational and individual level. Within an attachment-informed culture, services may act as ‘organisational caregivers’ that promote continuity of care, independence and stronger clinical relationships. In turn, this may foster more inclusive, responsive and resilient mental healthcare systems that prioritise patients’ needs and empowerment.

Mental healthcare has been the object of a significant evolution over the past half a century, characterised by developments in research, funding and services in many parts of the world.^1^ This process was arguably spearheaded by two major theoretical shifts: the development of the biopsychosocial model to understand mental health difficulties, and the recovery model to guide their treatment. First introduced by George Engel as ‘a challenge for biomedicine’ to look beyond biological factors,^2^ the biopsychosocial model was the first to place a strong emphasis on the interaction between biological, psychological and social elements in shaping an individual's physical and mental health. This promoted a novel person-centred approach that led to the development of the recovery model of treatment – one that focused primarily on resilience and quality of life rather than only symptom control.^3^

For traditional psychiatry this shift carried two important challenges. On one side, it meant recognising that the biomedical model – which conceptualises mental health difficulties as ‘brain diseases’^4^ – was not the only explanation for mental disorders. On the other, it also meant looking beyond reductionist concepts, such as relapse, remission and symptom profiles, to acknowledge the importance of recovery informed by people's social, environmental, spiritual and vocational needs^3,5^ – all of which are essential for a fulfilling and meaningful life with or without mental health difficulties.^6^

Today, despite wide international differences, contemporary psychiatry has generally embraced a biopsychosocial model, and mental health services tend to be more aware of the basic psychosocial factors potentially affecting their patients.^7^ However, this process appears to be far from complete. Common mental health difficulties (e.g. low mood, anxiety) in conditions that affect the brain, such as neurodegenerative diseases, are still primarily interpreted through a biomedical, pathologising lens.^8–10^ More systemic approaches – which would allow wider social determinants of poor mental as well as physical health such as culture, education, housing and poverty to be addressed^11,12^ – are also overlooked in many mental health services.^12–14^

Despite the efforts toward holistic and person-centred approaches made in the second half of the 20th century, it has been argued that contemporary psychiatric provision has instead gradually progressed toward reductionism.^15,16^ This process is believed to have been driven mostly by strict and overzealous evidence-based approaches, which tend to overlook contextual factors and may be unable to address complex physical and mental health needs.^16^ When taken to the extreme, this can lead healthcare policies of governments in countries such as the USA to be built on an evidence-based practice, benefiting insurance and pharmaceutical companies more than patients and practitioners.^16^

Paradoxically, the adoption of a reductionist approach can also lead to the implementation of heuristic or pragmatic models of care with limited evidence base. A prime example of this has been the implementation of psychiatric in-patient models divided between acute and community care, each provided by different specialist teams.^17^ Shaped around other medical specialities and initially developed to improve the quality and logistics of care, the benefits of these models for mental healthcare are yet to be determined. A number of studies have suggested that people using in-patient services are more satisfied when the same psychiatrist is responsible for their care in both the in-patient and community settings, and that trust and positive therapeutic relationships are essential for patients’ satisfaction.^17,18^

## The pitfall of fragmentation

Because managing healthcare systems is beyond doubt a complex task, a tendency toward reductionism may be seen as a way of understanding and managing very complex realities in a manner that feels controllable.^19,20^ However, over time, reductionist approaches such as functional models often appear to have failed to consider the unintended consequence of creating a fragmented system.^21^ As contemporary mental healthcare systems keep placing significant emphasis on short-term care, brief interventions and specific outcomes, an increasing number of people may be at risk of falling through the system's cracks by not fitting the complex criteria for specialist services and interventions. This may paradoxically result in higher rates of hospital admission and community interventions, and, ultimately, greater economic burden for healthcare systems and governments.

A number of psychiatric services have also been traditionally developed around specific conditions or disorders, rather than the very people accessing them. As a result, individuals are often required to describe their circumstances, including sensitive issues, multiple times across different services – sometimes only to be signposted elsewhere or sent back to original referrer. Unsurprisingly, these fragmented processes generate a significant delay before effective help is delivered, and, especially during a crisis, may exacerbate low mood and anxiety and perpetuate pre-existing issues because they fail to consider patients’ attachment styles and needs.

Initially developed by John Bowlby^22^ and later expanded upon by Mary Ainsworth,^23^ attachment theory explores the profound emotional bonds formed between individuals and their caregivers. Its fundamental assumption is that children's early interactions with primary caregivers shape their beliefs about themselves, others and relationships. This process results in the development of different attachment styles – categorised as secure, anxious–ambivalent, avoidant and disorganised – which can have profound effects on lifelong patterns of behaviour and interpersonal relationships.^22,23^

An example of the effect of fragmentation on attachment styles was offered by Adshead,^24^ who highlighted that the way current services are designed may promote insecure and ambivalent attachment in people, particularly because of repeated transfers and referrals across specialist teams – often leaving little to no agency to patients themselves.^24^ This is especially relevant when working with serious mental illness (SMI), since a report published by the Schizophrenia Commission highlighted the fragmentation of services as a potential perpetuating factor for psychosis, as well as the reason why patients can feel ‘as if on a factory production line’ rather than properly looked after.^25^ These observations were often placed within the context of a broken and demoralised system with lack of person-centred approaches, family members’ involvement and, once again, continuity of care.

On the other hand, the same report praised the approach of other teams working with SMI, such early intervention in psychosis (EIP), which promoted active engagement of patients and their social networks, and encouraged independence and informed choices – all factors which may be seen as attachment-informed. The recent National Institute for Health and Care Excellence guidelines for EIP in the UK^26^ stressed the importance of such factors, recommending that care coordinators establish a therapeutic relationship with patients and their family, ideally from the first point of contact to the final discharge.

In another example related to SMI, continuity of care (and thus secure therapeutic attachment) was also a major focus of the development of the Trieste model of mental healthcare, which has been hailed by the World Health Organization as one of the most progressive in the world.^27,28^ Spearheaded by Italian psychiatrist and neurologist Franco Basaglia (1924–1980) – the principal proponent of the eponymous law that dismantled asylums and reformed the psychiatric system in Italy – this model saw lack of continuity of care in people with SMI as contributing to chronicity and relapses, with a great emphasis on offering flexibility to maintain a relationship with the patient and to avoid de-socialisation and abandonment.^27,29,30^

The negative impact of fragmentation in terms of clinical outcomes is also no mystery. When considering SMI, the lack of attachment-informed factors such as continuous care and secure therapeutic relationships have been linked with poorer outcomes in people with schizophrenia.^31^ Perhaps even more worryingly, however, such a link is likely to be found in all mental health conditions where the continuity of care is similarly poor. Over-reliance on specialist care has been highlighted as a potential contributing factor, with examples where the higher the number of community teams involved with a patient, the worse the clinical outcomes.^31^ Other authors have also emphasised how patients may be significantly affected by the behaviour of clinicians, describing an ‘efficacy paradox’, whereby the therapeutic relationship had greater influence on clinical outcomes than the pharmacological treatment that was being tested^32^ or even the specific psychotherapeutic model adopted.^33^

## Shifting paradigms

The current limitations of how mental healthcare is organised have led some clinicians to look at different paradigms to deliver safer and more effective mental health interventions.^34^ However, although there have been numerous attempts to change the way of delivering services or challenging the current way of thinking, few lasting results have been observed on healthcare institutions. For example, Barker^35^ proposed the Tidal model of care, with emphasis on metaphors and personal narratives to promote recovery. This has shown to be especially helpful in promoting sense of self and connectedness in people with SMI where continuous care is paramount, such as forensic patients^36^ and psychiatric populations with addictions.^37^

Sweeney et al^38^ instead highlighted the need for trauma-informed mental health services, which are derived from the notion that trauma is widespread among people living with mental health difficulties as well as in society in general. These promote a switch from a narrative based on asking ‘What is wrong with you?’ to a less blaming and pathologizing, ‘What happened to you?’,^39^ which is intended to result in a solution-focused approach. The re-traumatising processes of some of the current healthcare systems, in particular within in-patient settings, are what these approaches aim to avoid, with a focus on recovery and growth. A further attempt at changing perspectives may also be identified in the model of evolutionary psychiatry, which incorporates factors related to the history of human evolution (phylogenesis) and the development of individual human beings (ontogenesis) in the aetiology of mental health difficulties.

Although the models above have certainly contributed to a better understanding of healthcare challenges and offered alternative ways of conceiving service delivery, they have so far appeared to be unable to set clear principles and build a robust theoretical framework for the development of services that allow for a secure base, prompt responsiveness and relational continuity. For example, because of their strong focus on theoretical evolutionary meanings, the principles of evolutionary psychiatry have often been the object of speculation with regards to their effective translational value for clinicians.^40^

Furthermore, although often portrayed as ‘paradigm shifts’, such models do not represent a real shift according to the cycle of scientific revolution defined by Kuhn,^41^ which outlines distinct phases whereby science needs to go through a model drift, a model crisis and eventually, a model revolution. As many mental healthcare systems worldwide are certainly going through a model crisis phase, very few may ever reach the model revolution phase without a clear theoretical framework. The development of most mental health services over the past few decades has also appeared to rely on population-specific evidence bases and the allocation and management of finances. Although often essential, these driving processes hardly facilitate the adoption of robust and coherent theoretical frameworks that can help to understand human behaviour and emotional responses in everyday life, as well as in moments of crisis and suffering.

## Attachment and mental healthcare systems

As we have seen, reductionist approaches appear to have failed at addressing some of the fundamental issues affecting mental health services, particularly as complexity needs to be accepted as an intrinsic part of mental healthcare systems. The lack of clear guiding principles in mental health service development might also be partially responsible for the fragmentation of services and the lack of continuity of care, which, in turn, prevent the development of meaningful and effective relationships with clinicians as well as services as a whole.^24,25,31^ And, as mentioned above, this is particularly important when dealing with SMIs, which typically require longer-term care.^42^

A potential answer to this gap in guiding principles might be found in the abovementioned theory of attachment.^22,23^ With regards to service delivery, attachment theory suggests that caregivers should aim to provide a secure base to increase availability, flexibility, sensitivity and responsiveness to distress, while also intervening when needed to promote secure attachment.^22^ This appears even more relevant when, as highlighted by Adshead,^24^ attachment styles are conceived ‘as a biobehavioural stress management system that is activated whenever a person is distressed and vulnerable and seeks care or protection’. Attachment styles have largely been found to play a major role not only in the relationship with healthcare professionals, but also in treatment adherence and clinical outcomes across a wide range of mental and physical health conditions,^43,44^ and especially in individuals with SMI.^45^ Secure attachment facilitates the development of healthy psychological habits, effective emotion regulation and appropriate interpersonal support in times of distress, as well as the acquisition of adequate emotional lexicon and skills that enable individuals to identify and describe their feelings.^24^ It also promotes the development of autonomy, resilience and healthy interpersonal relationships, which, in turn, influence treatment adherence and long-term health outcomes.^24^

Nonetheless, as mentioned, the current organisation of mental health services does not necessarily favour secure attachment styles and continuity of care. The current emphasis on short-term treatments with insufficient time to establish a relationship that is safe and secure in attachment terms may promote insecure attachment, with obvious negative consequences for individuals.^24^ Access to support is also often described as difficult, and rigid inclusion criteria and a large number of teams or specialisms provide fragmented experiences of care.^31^ Similarly, Beale^46^ described how some mental healthcare systems appear to be designed either to coerce or to exclude, and how excluding patients from services has become common practice among clinicians, who think that it is clinically justified to do so. This, along with lack of resources and a risk-averse culture, may lead to systematic exclusion and iatrogenic harm in patients, as well as moral injury and erosion of compassion in mental health professionals.^46^

The suggestion that attachment theory may inform the design of mental health services is not new. A little less than a decade ago, Bucci et al^47^ reviewed the literature regarding attachment theory within mental health policy and service development, and concluded that it may indeed represent a viable theoretical framework. They identified eight key themes to guide the development of attachment-informed services, including service policies and evaluation, referrals, assessments and formulations, interventions, and support for staff and informal carers. The general lack of evidence on the adoption of attachment theory, as well as the lack of evaluation of services based on the ability to meet attachment needs, were also highlighted.^47^

A need to move from specialist to more generalist care to develop attachment-informed services has also been identified previously. Crossley and Lepping^48^ described the role confusion that current specialist-focused systems may create, and emphasised how more generalist systems may act with safety and best interest in mind rather than focusing on targeted changes, and may prevent patients from becoming lost in the system by attempting to reproduce secure attachment. A similar concept has been supported by Laugharne et al,^49^ who defended generalist services as a place where continuity of care and therapeutic relationships are easier to establish.

With regards to attachment-informed systems, a virtuous example is perhaps represented by Sweden's ‘Esther’ model. Developed in the late 1990s from the negative experience of a fictional elderly patient (‘Esther’),^50^ its overarching principle was a shift from narrow, organisation-centred views – focused on the best course of action for a specific unit or department – to a more patient-centred approach, summarised by the question ‘What is best for Esther?’.^51^ The Esther model has so far been successfully implemented for individuals with complex health needs in some places across different countries, including the UK^52^ and Singapore.^53^ There, the model created a system in which healthcare organisations provide care in a collaborative, organised and supportive fashion^54^ by focusing on ‘what the individual wants, not what professionals think they should have’.^52^ This level of cohesion is believed to simulate the stability of a nurturing and healthy parental relationship, and thus, a stable and safe base for individuals navigating the system and receiving interventions. Ultimately, the adoption of the principles required to promote a secure attachment in the Esther model may represent a catalyst for recovery when caring for people with SMI and other conditions requiring longer-term care (e.g. elderly patients with complex needs^50^), in whom attachment is known to play a crucial role with regards to functioning.^45^

The same determining factors of secure attachment – such as security, respect, responsiveness, negotiation, promotion of human rights and autonomy – also underpin the good outcomes of the abovementioned model of mental healthcare in Trieste.^29^ However, the principles of the Trieste model are not bound by a theoretical framework, which may be one of the reasons why it has been historically difficult to replicate on a large scale, both within Italy as well as elsewhere in the world.^27,30^

## A few ideas for the future

Despite receiving previous attention,^47^ and besides few sporadic examples such as the Esther and Trieste models,^30,54^ attachment theory still struggles to find its way within the development of mental healthcare services. It is perhaps easier to think of attachment at the individual level, with a focus on how the attachment style of patients affects presentations and how the attachment style of healthcare providers affects responses. However, it could be argued that mental healthcare systems as a whole can be conceived of ‘organisational caregivers’ within attachment theory, thereby focusing on whether their organisational climate promotes secure levels of attachment for both patients and staff.

The quality and functions of an effective individual caregiver – such as availability, proactiveness and responsiveness – need to be translated into service delivery as accessible, proactive and continuous care at an organisational level. Mental health services may also need to challenge the current reductionist trends and move toward more generalist, inclusive, responsive and ‘defragmented’ models of care characterised by less stringent inclusion criteria. For example, barriers identified in current referral systems would need to be challenged, minimised and possibly eliminated in a timely fashion, including issues such as unhelpful financial management processes.

The meaning of multiagency and multidisciplinary work should also be invested with renewed significance, since the flexibility and responsiveness of both individual interventions and services as a whole should be at the centre of any type of development. The continuous collaboration between psychiatrists, clinical psychologists and other mental health professionals should be encouraged further – not only to ensure the delivery of effective integrated models of care, but also to promote the adoption of biopsychosocial and systemic approaches in mental health areas where strong pathologising narratives still persist.^8–10^

Finally, but importantly, a potential misconception should be clarified, i.e. that attachment-based services will create dependence. A clear distinction should be made between offering attachment-informed services and promoting dependence, as the main goal of mental healthcare should remain the promotion of independence and self-care.^47^ Where mental health services truly promote a secure attachment by creating a safe place, independence should follow naturally.

## Conclusions

Attachment theory may represent a viable and effective theoretical framework when designing and managing mental healthcare systems, and especially with populations requiring longer-term care, such as people with SMI. First, its principles should be applied to the system as a whole, making sure that organisational climates and models of care address patients’ need for safety, responsiveness, consistency and openness, while avoiding the pitfalls of reductionism and fragmentation and promoting continuous care at their core. The need for generalist models of care should be considered against the current trend of specialist services, and audits should be carried out on how attachment principles are applied.

Second, a cultural change is perhaps warranted in the way healthcare professionals perform their functions. Attachment theory provides clear principles on how humans function and how to respond to their needs. When applied to mental healthcare systems, this should be at the heart of multiagency and multidisciplinary work that considers the needs of patients, their families and healthcare staff in equal measure.

Ultimately, the development of attachment-informed mental health systems as ‘organisational caregivers’ should aim at facilitating independence and resilience by enabling individuals to make informed and collaborative decisions on the interventions they need, and develop lifelong skills to manage their mental health.

## About the authors

**Alberto Salmoiraghi** is a consultant psychiatrist and Medical Director for the Mental Health and Learning Disabilities Division in North Wales, Betsi Cadwaladr University Health Board, Bangor, UK; an honorary senior lecturer at Bangor University, Bangor, UK; and visiting professor at Wrexham University, Wrexham, UK. **Nicolò Zarotti** is an academic and clinical psychologist with the Department of Clinical Neuropsychology at the Manchester Centre for Clinical Neurosciences (MCCN), Salford Royal NHS Foundation Trust, Salford, UK; and the Division of Health Research at Lancaster University, Lancaster, UK.

## Data Availability

Data availability is not applicable to this article as no new data were created or analysed in this study.

## References

[1] Jones K. A History of the Mental Health Services. Taylor & Francis, 2023.

[2] Engel GL. The need for a new medical model: a challenge for biomedicine. Science (1979) 1977; 196: 129–36. 10.1126/science.847460 847460

[3] Jacob K. Recovery model of mental illness: a complementary approach to psychiatric care. Indian J Psychol Med 2015; 37: 117–9. 25969592 10.4103/0253-7176.155605 PMC4418239

[4] Deacon BJ. The biomedical model of mental disorder: a critical analysis of its validity, utility, and effects on psychotherapy research. Clin Psychol Rev 2013; 33: 846–61. 23664634 10.1016/j.cpr.2012.09.007

[5] Stylianidis S, Lavdas M, Markou K, Belekou P. The recovery model and modern psychiatric care: conceptual perspective, critical approach and practical application. In Social and Community Psychiatry: Towards a Critical, Patient-Oriented Approach (ed S Stylianidis): 145–65. Springer International Publishing, 2016.

[6] Substance Abuse and Mental Health Services Administration (SAMHSA). *Recovery and Recovery Support.* SAMHSA, 2024 (https://www.samhsa.gov/find-help/recovery).

[7] Tripathi A, Das A, Kar S. Biopsychosocial model in contemporary psychiatry: current validity and future prospects. Indian J Psychol Medicine 2019; 41: 582–5. 10.4103/IJPSYM.IJPSYM_314_19 PMC6875848 31772447

[8] Zarotti N, Dale M, Eccles FJR, et al. More than just a brain disorder: a five-point manifesto for psychological care for people with Huntington's disease. J Pers Med 2022; 12: 64. 35055379 10.3390/jpm12010064 PMC8780585

[9] Simpson J, Eccles FJ, Zarotti N. *Extended Evidence-Based Guidance on Psychological Interventions for Psychological Difficulties in Individuals with Huntington's Disease, Parkinson's Disease, Motor Neurone Disease, and Multiple Sclerosis*. Lancaster University, 2021 (https://zenodo.org/records/4593883).

[10] Dale M, Wood A, Zarotti N, et al. Using a clinical formulation to understand psychological distress in people affected by Huntington's disease: a descriptive, evidence-based model. J Pers Med 2022; 12: 1–19. 10.3390/jpm12081222 PMC9332789 35893316

[11] Marmot M. Health equity in England: the Marmot review 10 years on. BMJ 2020; 368: m693. 10.1136/bmj.m693 32094110

[12] Cohen M. A systemic approach to understanding mental health and services. Soc Sci Med 2017; 191: 1–8. 28881215 10.1016/j.socscimed.2017.08.037

[13] Huang Y, Li Y, Haun MW, et al. Systemic therapy for children and adolescents with depression: a systematic review and meta-analysis. Curr Psychol 2024; 43: 3355–67.

[14] Pinquart M, Oslejsek B, Teubert D. Efficacy of systemic therapy on adults with mental disorders: a meta-analysis. Psychother Res 2016; 26: 241–57. 25032487 10.1080/10503307.2014.935830

[15] Roache R. Psychiatry's problem with reductionism. Philos Psychiatry Psychol 2019; 26: 219–29.

[16] Webb L. The recovery model and complex health needs: what health psychology can learn from mental health and substance misuse service provision. J Health Psychol 2012; 17: 731–41. 22021273 10.1177/1359105311425276

[17] Bird VJ, Giacco D, Nicaise P, et al. In-patient treatment in functional and sectorised care: patient satisfaction and length of stay. Br J Psychiatry 2018; 212: 81–7. 29436328 10.1192/bjp.2017.20

[18] Laugharne R, Pant M. Sector and functional models of consultant care: in-patient satisfaction with psychiatrists. Psychiatrist 2012; 36: 254–6.

[19] Plsek PE, Greenhalgh T. Complexity science: the challenge of complexity in health care. BMJ 2001; 323: 625–8. 11557716 10.1136/bmj.323.7313.625 PMC1121189

[20] Dooris M, Farrier A, Froggett L. Wellbeing: the challenge of ‘operationalising’ an holistic concept within a reductionist public health programme. Perspect Public Health 2018; 138: 93–9. 28574301 10.1177/1757913917711204

[21] Australian Government Department of Health and Aged Care. *Australian Government Response to Contributing Lives, Thriving Communities – Review of Mental Health Programmes and Services*. Australian Government, 2015 (https://www.health.gov.au/resources/publications/australian-government-response-to-contributing-lives-thriving-communities-review-of-mental-health-programmes-and-services?language=en).

[22] Bowlby J. Attachment and Loss. Random House, 1969.

[23] Ainsworth MS. Infant–mother attachment. Am Psychol 1979; 34: 932–7. 517843 10.1037//0003-066x.34.10.932

[24] Adshead G. Security of mind: 20 years of attachment theory and its relevance to psychiatry. Br J Psychiatry 2018; 213: 511–3. 30113289 10.1192/bjp.2018.104

[25] Schizophrenia Commission. *The Abandoned Illness: A Report by Schizophrenia Commission*. Schizophrenia Commission, 2012 (https://www.rethink.org/media/2637/the-abandoned-illness-final.pdf).

[26] NHS England. *Implementing the Early Intervention in Psychosis Access and Waiting Time Standard*, Version 3. NHS England, 2023 (https://www.england.nhs.uk/wp-content/uploads/2023/03/B1954-implementing-the-early-intervention-in-psychosis-access-and-waiting-time-standard.pdf).

[27] Portacolone E, Segal SP, Mezzina R, et al. A tale of two cities: the exploration of the Trieste public psychiatry model in San Francisco. Cult Med Psychiatry 2015; 39: 680–97. 25998781 10.1007/s11013-015-9458-3

[28] World Health Organization (WHO). Mental Health in Europe: Stop Exclusion, Dare to Care. WHO, 2001 (https://iris.who.int/bitstream/handle/10665/348009/WHO-EURO-2001-3970-43729-61519-eng.pdf?sequence=1&isAllowed=y).

[29] Mezzina R, Vidoni D. Beyond the mental hospital: crisis intervention and continuity of care in Trieste. a four year follow-up study in a community mental health centre. Int J Soc Psychiatry 1995; 41: 1–20. 7622336 10.1177/002076409504100101

[30] Mezzina R. Community mental health care in Trieste and beyond: an ‘open door-no restraint’ system of care for recovery and citizenship. J Nerv Ment Dis 2014; 202: 440–5. 24840089 10.1097/NMD.0000000000000142

[31] MacDonald A, Adamis D, Craig T, et al. Continuity of care and clinical outcomes in the community for people with severe mental illness. Br J Psychiatry 2019; 214: 273–8. 31012407 10.1192/bjp.2018.261

[32] McQueen D, Cohen S, John-Smith PS, et al. Rethinking placebo in psychiatry: how and why placebo effects occur. Adv Psychiatr Treat 2013; 19: 171–80.

[33] Norcross JC, Lambert MJ. Evidence-based psychotherapy relationship: the third task force. In Psychotherapy Relationships That Work: Evidence-Based Therapist Contributions (eds JC Norcross, MJ Lambert): 1–23. Oxford University Press, 2019.

[34] The Lancet Psychiatry. Shifting paradigms. Lancet Psychiatry 2022; 9: 525. 35717950 10.1016/S2215-0366(22)00210-3

[35] Barker P. The tidal model. the healing potential of metaphor within a patient's narrative. J Psychosoc Nurs Ment Health Serv 2002; 40: 42–50. 10.3928/0279-3695-20020701-12 12136515

[36] Cook NR, Phillips BN, Sadler D. The tidal model as experienced by patients and nurses in a regional forensic unit. J Psychiatr Ment Health Nurs 2005; 12: 536–40. 16164503 10.1111/j.1365-2850.2005.00872.x

[37] Kusdemir S, Oudshoorn A, Ndayisenga JP. A critical analysis of the tidal model of mental health recovery. Arch Psychiatr Nurs 2022; 36: 34–40. 35094823 10.1016/j.apnu.2021.10.012

[38] Sweeney A, Filson B, Kennedy A, et al. A paradigm shift: relationships in trauma-informed mental health services. BJPsych Adv 2018; 24: 319–33. 30174829 10.1192/bja.2018.29 PMC6088388

[39] Winfrey O, Perry B. What Happened to You?: Conversations on Trauma, Resilience, and Healing. Pan Macmillan, 2021.

[40] Nesse RM. Evolutionary psychiatry: foundations, progress and challenges. World Psychiatry 2023; 22: 177–202. 37159362 10.1002/wps.21072 PMC10168175

[41] Kuhn TS. The Structure of Scientific Revolutions. University of Chicago Press, 1962.

[42] Wiersma D. Needs of people with severe mental illness. Acta Psychiatr Scand 2006; 113: 115–9. 10.1111/j.1600-0447.2005.00728.x 16445493

[43] Palmer Kelly E, Tsilimigras DI, Hyer JM, et al. Understanding the use of attachment theory applied to the patient-provider relationship in cancer care: recommendations for future research and clinical practice. Surg Oncol 2019; 31: 101–10. 31622916 10.1016/j.suronc.2019.10.007

[44] Klest B, Tamaian A, Boughner E. A model exploring the relationship between betrayal trauma and health: the roles of mental health, attachment, trust in healthcare systems, and nonadherence to treatment. Psychol Trauma 2019; 11: 656–62. 30896222 10.1037/tra0000453

[45] Pearse E, Bucci S, Raphael J, et al. The relationship between attachment and functioning for people with serious mental illness: a systematic review. Nord J Psychiatry 2020; 74: 545–57. 32692588 10.1080/08039488.2020.1767687

[46] Beale C. Magical thinking and moral injury: exclusion culture in psychiatry. BJPsych Bull 2022; 46: 16–9. 34517935 10.1192/bjb.2021.86 PMC8914811

[47] Bucci S, Roberts NH, Danquah AN, et al. Using attachment theory to inform the design and delivery of mental health services: a systematic review of the literature. Psychol Psychother 2015; 88: 1–20. 24729543 10.1111/papt.12029

[48] Crossley D, Lepping P. Role confusion, values-based practice and the demise of the generalist. Psychiatr Bull 2009; 33: 7–9.

[49] Laugharne R, Thompson M, Srivastava A, et al. Getting a balance between generalisation and specialisation in mental health services: a defence of general services. BJPsych Bull 2018; 42: 229–32. 30012234 10.1192/bjb.2018.52 PMC6465214

[50] Gray BH, Winblad U, Sarnak DO. Sweden's Esther Model: Improving Care for Elderly Patients with Complex Needs. The Commonwealth Fund, 2016 (https://www.commonwealthfund.org/publications/case-study/2016/sep/swedens-esther-model-improving-care-elderly-patients-complex-needs).

[51] Vackerberg N, Andersson AC, Peterson A, et al. What is best for Esther? A simple question that moves mindsets and improves care. BMC Health Serv Res 2023; 23: 873. 10.1186/s12913-023-09870-1 PMC10433680 37592279

[52] Lee S, Vibhuti M, Therkildsen T. Sustainable person-centred communities design and practice. In International Practice Development in Health and Social Care (eds K Manley, V Wilson, C Øye): 39–51. Wiley Blackwell, 2021.

[53] Lim ELP, Khee GY, Thor J, et al. How the Esther network model for coproduction of person-centred health and social care was adopted and adapted in Singapore: a realist evaluation. BMJ Open 2022; 12: e059794. 10.1136/bmjopen-2021-059794 PMC9791430 36564117

[54] Institute for Healthcare Improvement. Improving Patient Flow: The Esther Project in Sweden. Institute for Healthcare Improvement, 2024 (https://staff.ihi.org/resources/Pages/ImprovementStories/ImprovingPatientFlowTheEstherProjectinSweden.aspx#).

